# Effects of Endotoxin and Psychological Stress on Redox Physiology, Immunity and Feather Corticosterone in Greenfinches

**DOI:** 10.1371/journal.pone.0067545

**Published:** 2013-06-21

**Authors:** Richard Meitern, Elin Sild, Mari-Ann Lind, Marju Männiste, Tuul Sepp, Ulvi Karu, Peeter Hõrak

**Affiliations:** 1 Department of Zoology, Institute of Ecology and Earth Sciences, Tartu University, Tartu, Estonia,; 2 Molecular Ecology and Evolution Lab, Department of Biology, Lund University, Lund, Sweden; University of Milan, Italy

## Abstract

Assessment of costs accompanying activation of immune system and related neuroendocrine pathways is essential for understanding the selective forces operating on these systems. Here we attempted to detect such costs in terms of disruption to redox balance and interference between different immune system components in captive wild-caught greenfinches (*Carduelis chloris*). Study birds were subjected to an endotoxin-induced inflammatory challenge and temporary exposure to a psychological stressor (an image of a predator) in a 2*2 factorial experiment. Injection of bacterial endotoxin resulted in up-regulation of two markers of antioxidant protection – erythrocyte glutathione, and plasma oxygen radical absorbance (OXY). These findings suggest that inflammatory responses alter redox homeostasis. However, no effect on markers of oxidative damage to proteins or DNA in erythrocytes could be detected. We found no evidence that the endotoxin injection interfered with antibody production against *Brucella abortus* antigen or the intensity of chronic coccidiosis. The hypothesis of within-immune system trade-offs as a cost of immunity was thus not supported in our model system. We showed for the first time that administration of endotoxin can reduce the level of corticosterone deposited into feathers. This finding suggests a down-regulation of the corticosterone secretion cascade due to an endotoxin-induced immune response, a phenomenon that has not been reported previously. Exposure to the predator image did not affect any of the measured physiological parameters.

## Introduction

Virtually all organisms are exposed to a wide range of potential pathogens and thus have evolved complex physiological responses, including innate and acquired immunity, to resist and eliminate infections [1]. However, using the immune system can be costly, as revealed by studies demonstrating adverse effects of immune defence activation on different components of fitness and various physiological indicators of well-being in hosts [2]–[5]. Assessment of such costs is essential for understanding the selective forces operating on immune responses [6]–[8].

The costs of using immune responses can be roughly divided into energetic [7], immunopathological [9] and those resulting from cross-regulation between different components of the immune system, such that responses against one type of pathogen render a host susceptible to other types of pathogen [10], [11]. The current study focuses on the two latter types of cost in relation to endotoxin-induced inflammatory response in captive wild-caught greenfinches (*Carduelis chloris*).

Immunopathology, the damage to a host caused by immune responses *per se*, is implicated in the severity of many of the world's major human diseases; however, its role in wild animal diseases has been little studied [12]. One of the most potent sources of pathological damage exerted by the immune system upon its host is the production of reactive oxygen and nitrogen species by phagocytic cells during an inflammatory response [6], [12]. These reactive species are not pathogen-specific and can also damage the host if it's protective and/or repair functions are compromised or fail. This may cause oxidative stress (OS), defined here as ‘a disturbance in the pro-oxidant/antioxidant balance in favour of the oxidants, leading to a disruption of redox signalling and control and/or molecular damage’ [13]. However, despite OS being regularly cited as a major cause of immunopathology [12], evidence for the claim appears contradictory [14].

Cross-regulation between different components of the immune system is another important source of immunity costs and may also relate to generation of immunopathological damage [11]. To date, most of the evidence concerning trade-offs between different components of the immune system in the context of immunoecological research originates from studies of mammals [15]. Evidence for similar patterns in birds has started to accumulate but originates mostly from studies of poultry [16], [17] (but see [18], [19]).

An ecologically relevant method for examining physiological tradeoffs within the immune system is to compare the responses of stressed and unstressed animals [20]. Stress, defined here as a ‘physiological and behavioural state engaged to endure, avoid, or recover from an aversive stimulus or condition’ [21] is tightly integrated with immune function in vertebrates. Some components of immune responses are enhanced when confronted by certain acute stressors, such as encounters with predators or aggressive conspecifics, in order to prepare an organism for potential invasion of pathogenic microbes in the case of wounding [22]. However, other types of stressors and chronic stress in general rather appear immunosuppressive [21], [23], [24].

To study the effects of immune system activation on parameters of oxidative status and other components of immunity, we performed an experiment with 66 wild-caught captive greenfinches. We adopted the approach of inducing an inflammatory response by administering bacterial lipopolysaccharide (LPS), which is used as a model to mimic OS associated with injury and disease in biomedicine and poultry science [25] but also increasingly in ecological studies [26]. In a 2*2 factorial design, approximately half of the birds were injected with LPS and/or temporarily exposed to an image of a predator in order to impose psychological stress. We then assessed two markers of antioxidant protection (Total Antioxidant Capacity, TAC, and Oxygen Radical Absorbance, OXY), individual antioxidants (glutathione and uric acid) and two indicators of oxidative damage (protein carbonyls, a marker of protein oxidation, and DNA damage, assessed in single cell gel electrophoresis - comet assay). We also recorded the effects of treatments on body mass and total plasma protein content as indicators of general physiological condition. To assess the effects of treatments on parameters of immune function, we monitored changes in chronic coccidian infection intensity and antibody production in response to immunisation with *Brucella abortus* (BA) antigen. In order to assess the potentially stressful effects of both treatments on our study objects, we measured the corticosterone content of tail feathers grown during the experiment.

We predicted that (1) if induction of inflammatory response causes oxidative stress, we should detect higher levels of oxidative damage among LPS-injected birds as compared to saline-injected controls on the basis of elevated protein carbonyls and DNA damage. Similarly, we anticipated that (2) exposure to a stressful stimulus (an image of a predator) might induce oxidative damage [27], [28]–[30]. (3) If higher levels of antioxidant capacity and individual antioxidants reflect beneficial redox state, these should decrease in LPS and predator-image-exposed birds. Alternatively, if LPS injection induces OS that leads to protective up-regulation of antioxidant defences, antioxidant levels may increase in response to LPS and stress treatments [31], [32]. (4) We had no directional predictions with respect of the effects of LPS injection on our measures of immunity. Assuming that mounting an inflammatory response to LPS usurps resources that could be invested into other components of immunity [33], one might expect LPS treatment to reduce the antibody response to BA and increase the intensity of chronic coccidiosis. On the other hand, synergistic interactions between different immune system components are also possible [34], [35], such that priming an immune system with LPS might also potentiate immune responses to other antigens [36]. We predicted that (5) total plasma protein levels should increase in response to immune challenge if synthesis of positive acute phase proteins exceeded protein catabolism [37]. Alternatively, total plasma proteins might decrease due to inflammation-induced catabolism [38]. We further predicted that (6) both treatments would induce a decline in body mass and (7) elevate feather corticosterone due to inflammation- and/or stress-induced secretion of corticosterone [37], [39], [40]. Finally, we predicted that if the predator image exposure was sufficiently stressful in our model system, (8) all physiological effects of LPS injection should be boosted among birds exposed to the image [41]. Because physiological responses to immune system activation have been shown to depend on age [42], [43], we also tested whether yearling and older greenfinches respond differently to LPS treatments.

## Methods

### Study protocol

Male wild greenfinches were captured in mist-nets at bird feeders in a garden in the city of Tartu, Estonia (58° 22′ N; 26° 43′ E) on 2nd, 3rd and 9th January 2012. The birds were housed indoors in individual cages (27×51×55 cm) with sand-covered floors in a single room where they had visual contact with their neighbours. The average temperature in the aviary during the experiment was 14.2±1.5 (SD) ^o^C and average humidity was 43±9 (SD)%. The birds were supplied *ad libitum* with sunflower seeds and tap water and were exposed to a natural day-length cycle using artificial lighting by luminophore tubes. They were released back to their natural habitat on 9th March 2012. The study was conducted under license from the Estonian Ministry of the Environment (Licence # 1–4.1/11/100, issued on 23rd March 2011), and the experiment was approved by the Committee of Animal Experiments at the Estonian Ministry of Agriculture (decision # 95, issued on 17th January 2012).

**Figure 1:**
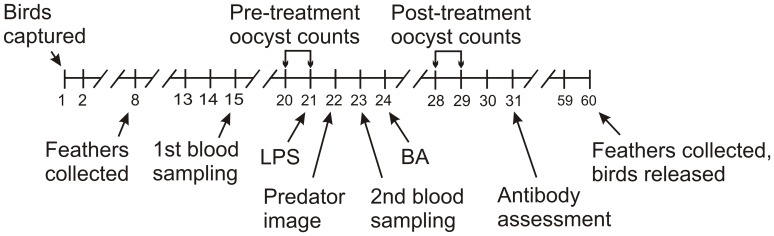
Timeline of the experiment. Day 1 = 10th January. ‘LPS’ and ‘BA’ stand for bacterial lipopolysaccharide and *Brucella abortus* antigen injection, respectively. ‘Predator image’ stands for exposing birds to an image of an owl (Fig. 2), mounted on their food cups.

The timeline of the experiment is shown in Fig. 1. The birds were divided into four approximately equal-sized groups on the basis of similar age composition (yearlings vs older, determined on the basis of plumage characteristics) and body mass, recorded on 24 January. The age composition of the treatment groups is given in Table S1. On the evening of the 21^st^ day of the experiment (30th January), after the lights had switched off, 31 birds received an injection of 0.1 mg *E. coli* LPS (strain 055:B5, Sigma L2880) in 40 µL sterile isotonic saline into the pectoralis muscle. The dose of LPS was chosen to be identical to that used in a similar experiment in house sparrows (*Passer domesticus*) [44]. The remaining birds (35 individuals) received 40 µL isotonic saline injections.

**Figure 2:**
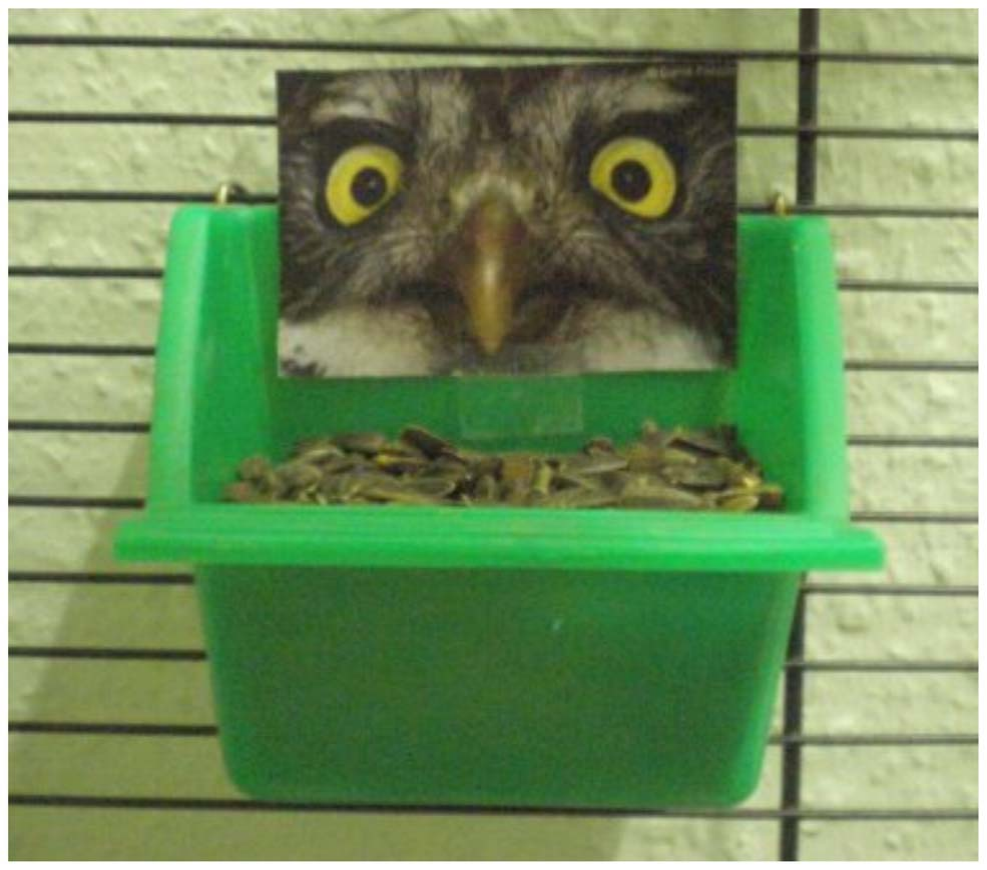
Image of predator, mounted on the food cup. On day 24 all of the birds were injected with 40 µL of killed *Brucella abortus* (BA) suspension in the pectoralis muscle (serum agglutination test antigen, Strain 99, product code RAA0054, Veterinary Laboratories Agency, Weybridge, UK). All birds were blood sampled on days 15 and 23 in order to record the effects of treatments on haematological parameters and in the morning of day 31 to collect serum samples for determination of anti-BA antibodies. The 38 h period between the LPS injection and the second blood sampling was chosen because we expected that ecologically relevant effects of inflammatory challenge on the studied physiological markers would last longer than one day. Blood sampling of birds took place in the mornings before the lights turned on. Other procedures, including coccidian oocyst collection, maintenance and BA injections, were performed in the evenings after the lights had turned off.

At the same time, food cups were removed from all cages and cage floors were completely cleaned in order to motivate the birds to feed when presented with a predator image the following morning. In the morning of day 22, the birds were kept without food for 2 h 40 min (the total length of the light period was 9 h 15 min). During the final 10 min of that period, the lights were turned off and the birds received their food cups, filled with approximately equal amount of sunflower seeds. Thirty-two birds (16 LPS-injected and 16 saline-injected) received an image of an owl (Fig. 2) attached to their food cups, as birds are known to be averse to pairs of eyes (e.g., [45]). The photo of *Glaucidium nanum* by Danté Fenolio was obtained from http://anotheca.com/wordpress/2009/09/02/working-in-chile-to-conserve-darwins-frogs/. Thereafter, the lights were turned on and the exposure of the owl image lasted for 83 min. At that point, the images were removed from the cups, and the birds continued their daily routine until the lights switched off at 17:15. The owl image was obviously perceived by the birds as threatening, as the average latency to feed was more than seven times longer among the image-exposed than the non-exposed birds (28.3±26.8 min vs 3.8±6.4 min; z = 4.8, P<0.0001, U-test). We chose not to use a non-threatening ‘control’ image for the birds that were not exposed to the owl image because the aim of the experiment was not to detect a response to a specific predator but to modify the context of physiological and behavioural measurements. From this perspective, there is no difference whether the birds perceived an image as a predator or just as a novel object.

Birds were sampled for coccidian (*Isospora* sp.) infection by faecal examination on days 20 – 21 (pre-treatment oocyst counts) and on days 28 – 29 (post-treatment oocyst counts). For collection of faecal samples, two sheets of A4 paper were placed on the sand bedding of cages 2 h before the lights turned off. After the lights had turned off, faeces were collected from the papers. Infection intensities were averaged over the pre- and post-treatment period. All birds appeared naturally infected. Infection intensities (number of oocysts per gram of faeces) of individual birds were quantified as described previously [46], [47].

### Blood analyses

Concentration of uric acid was determined from 5 µL plasma samples using the enzymatic colorimetric test with lipid clearing factor (uric acid liquicolor). Repeatability of uric acid concentration was 0.95 (F_9,10_ = 35.7, P <0.0001). Total plasma protein was determined from 5 µL samples using the biuret method. Repeatability of total plasma protein concentration was 0.90 (F_9,10_ = 19.2, P <0.0001). Both kits were manufactured by Human GmbH (Wiesbaden, Germany).

Plasma Total Antioxidant Capacity (TAC) was measured from 5 µL plasma samples according to the method described by Erel [48] with minor modifications as described by Sepp and colleagues [49]. The assay is based on the capacity of antioxidants in the solution to decolorize the ABTS^+^ (2, 2 – azinobis (3-ethylbenzothiazoline-6-sulfonate)) according to their concentrations and antioxidant capacities. The main contributors to TAC are plasma uric acid and free sulfhydryl groups of proteins [48], [49]. The results are quantified in mM Trolox (water soluble vitamin E analogue) equivalents. Repeatability of TAC was 0.96 (F_8,9_ = 44.0, P<0.0001) [49].

Plasma Oxygen Radical Absorbance (OXY) was measured using the OXY-adsorbent test (Diacron International, Grosseto, Italy) from 5 µL plasma samples, according to the manufacturer's instructions [50]. This test quantifies the ability of the plasma non-enzymatic antioxidant compounds to cope with the *in vitro* oxidant action of hypochlorous acid (HOCl; an endogenously produced oxidant). Concentrations of OXY are expressed as mM of HOCl neutralized. Repeatability of OXY was 0.79 (F_9,10_ = 8.3, P = 0.0007) [51]. Previous studies in birds, including greenfinches, have shown that OXY does not correlate with TAC [51].

Total glutathione (GSH) levels in erythrocytes were determined within a week after sampling following the approach of Galván and Alonso-Alvarez [52] and Rahman et al. [53] with modifications as previously described [54]. Results are given in μM per gram of pellet. Repeatability of GSH concentration was 0.91 (F_9,10_ = 22.1, P<0.0001) [54].

Determination of carbonyl level in proteins is used as an index of the extent of protein oxidative damage. The assay was carried out as previously described [55]. Briefly, after centrifugation, erythrocytes were washed twice with 0.9% sodium chloride and were centrifuged at 6700 g. A 5% erythrocyte suspension in 0.15 M NaCl 10 mM sodium phosphate buffer (pH 7.4) was then stored at −20°C until 200 µl of the haemolysate was mixed with 600 µl of 8 mM 2,4-dinitrophenylhydrazine in 3 M HCL. Samples were incubated for 50 min at 37°C in the dark and vortexed every 10 min. 600 µl of 25% (w/v) trichloroacetic acid was added to the tube, which was left on ice for 8 min and then centrifuged for 3 min at 6000 g to collect the protein precipitates. This pellet was washed using 200 µl 15% trichloroacetic acid and then washed twice with 800 µl of ethanol-ethyl acetate (1:1), (v/v). The final precipitate was dissolved in 300 µL 5M guanidine hydrochloride solution and incubated for 15 min at 37°C with mixing. Any insoluble materials were removed by repeated centrifugation. Carbonyl level was calculated from the peak absorbance of the spectra at 355–390 nm, using an absorption coefficient of 22000 M^−1^ cm^−1^. Results were expressed as μmol per mg of protein. The protein content of the sample was assessed using the Bio-Rad Protein Assay kit (Bio-Rad Laboratories, Inc., CA, USA) according to the manufacturer's instructions. Repeatability of protein carbonyl concentration was 0.65 (F_51,92_ = 6.2, P<0.0001).

The comet assay was performed using the Trevigen Comet Assay kit (Trevigen Inc., Gaithersburg, MD, USA) with modifications in electrophoresis conditions as indicated below. The alkaline version of the assay measures DNA strand breaks and alkali-labile sites, i.e. apurinic/apyrimidinic sites or baseless sugars, while the use of bacterial repair endonucleases enables detection of oxidized DNA bases (mainly 8-oxo-7,8-dihydroguanine (8-oxoGua)). In order to calculate the amount of oxidized DNA bases we simultaneously ran both the basic alkaline version of the comet assay (detects strand breaks and alkali-labile sites) and the same comet assay on cells incubated with a bacterial endonuclease (formamidopyrimidine DNA glycosylase; FPG). However, because the extent of DNA damage of FPG-incubated cells did not exceed the extent of DNA damage measured by the basic alkaline version of the assay, only the results obtained from the basic version of the assay appeared suitable for reporting [56].

DNA intactness in erythrocytes was evaluated according to the instructions in the Trevigen FLARE Assay kit manual, with slight changes in electrophoresis conditions. Briefly, immediately after sampling, 1 µL of whole blood was serially diluted in PBS (Ca^2+^ and Mg^2+^ free) to reach a concentration of roughly 1×10^5^ cells/ml. 10 µL of the obtained suspension was then mixed with 105 µL of molten LMAgarose (1% low-melting agarose, Trevigen) and 50 µL were immediately pipetted and evenly spread onto both wells of the comet slide. The slide was then incubated at 4°C in the dark for 60 min to accelerate gelling of the agarose disc and then transferred to prechilled lysis solution (Trevigen) with 10% DMSO for 4±1 (SD) h at 4°C. Next, the slides were washed three times for 10 min each in buffer (10 mM HEPES, 0.1 M KCl, 10 mM EDTA, 0.2 mg/ml bovine serum albumin, pH = 7.4) and then incubated in a humidity chamber at 37°C for between 30 and 45 min. For each slide, one well received 80 µL buffer containing 28 U/mL bacterial formamidopyrimidine DNA glycosylase (FPG, Sigma F3174), while the same amount of plain buffer was pipetted into the other well. After incubation, a denaturation step was performed in alkali solution (300 mM NaOH, 1 mM EDTA, pH = 13) at room temperature for 20 min, in the dark. The slide was then transferred to a prechilled alkaline electrophoresis solution pH = 13 (300 mM NaOH, 1 mM EDTA) and subjected to electrophoresis at 1 V/cm for 5 min in the dark at room temperature. At the end of the electrophoresis, the slides were washed two times for 10 min with distilled water, immersed in 70% ethanol at room temperature for 5 min and air dried at 37°C. DNA was stained with 50 µL of SYBR Green I dye (Trevigen, 1:10 000 in Tris–EDTA buffer, pH 7.5) for 20 min in the dark and immediately analyzed using an Olympus digital camera attached to an Olympus BX41 epifluorescence microscope.

For each slide well, 82±25 (SD) randomly chosen comets were analyzed using an Olympus BX41 epifluorescence microscope with an excitation filter of 470–490 nm and a barrier filter of 510–550 nm. Fluorescent images of single cells were captured at 100× magnification and images were scored for percentage of DNA in the tail using the Tritek CometScore™ Freeware v1.5 image analysis software. After scoring, the 30 most aberrant comets for each individual were excluded from the dataset. Repeatability of DNA damage (percentage of DNA in the tail in alkali-labile sites) was 0.85 (F_56;2893_ = 284, P<0.0001). Repeatability of average DNA damage among microscopic fields was 0.87 (F_115,231_ = 20.4, P<0.0001).

### Measurement of feather corticosterone

Wild-grown feathers (right outermost rectrix) were collected on day 8 of the experiment from each bird. Replacement feathers, grown during the experiment (lab-grown feathers) were collected on day 60, before the release of the birds. LPS/saline injections were performed 13 days after plucking the wild-grown feathers, when lab-grown replacement feathers were on average 8 mm long (13% of final length). Corticosterone from feathers was measured by radioimmunoassay (RIA) [57]–[59]. A methanol-based extraction technique was used to extract corticosterone from feathers. The calamus was removed and feather vanes minced into pieces of<5 mm^2^ with scissors. 10 mL of methanol (Sigma-Aldrich 34860N) was added and the samples placed in a sonicating water bath at room temperature for 30 min, followed by incubation at 50°C 300 rpm overnight in a shaker. The methanol was then separated from feather material by vacuum filtration, using #4 Whatman filter paper. The feather remnants, original sample vial and filtration material were washed with 3 mL of additional methanol; these washes were added to the original methanol extract. The methanol extract was placed in a 60°C water bath and subsequently evaporated in a fume hood under air. The extract residues were reconstituted in 0.55 ml PBS buffer (Sigma P4417) and frozen at -20°C until further analysis.

100 µL of anti-corticosterone Sigma-Aldrich C8784 (stock solution diluted 1:100 in 0.05 M Tris-HCl, pH 8, 0.1 M NaCl, 0.1% BSA, 0.1% sodium azide buffer) and 100 µL of tritiated (^3^H) corticosterone (Perkin Elmer, about 10000 cpm, in 0.05 M Tris-HCl, pH 8, 0.1 M NaCl, 0.1% BSA, 0.1% sodium azide buffer) were added to 200 µL of sample in duplicates and then incubated at +4°C overnight. Subsequently, 0.5 ml of dextran coated charcoal suspension (0.5% dextran Sigma 31390, 0.5% charcoal Sigma-Aldrich C3345 in 0.05 M Tris-HCl, pH 8, 0.1 M NaCl, 0.1% BSA, 0.1% sodium azide buffer, 0°C on magnetic stirrer) was added. Samples were incubated at +4°C for 10 min then centrifuged at +4°C at 2000 g for 10 min. Supernatants were removed and 0.5 mL of supernatant was transferred to a scintillation tube; 5 mL of scintillation cocktail (Optiphase ‘Hisafe’ 3, Perkin Elmer, USA) was added and samples were read on a Perkin Elmer Precisely Liquid Scintillation Analyser Tri-Carb 2800TR with 18 s count time. The assay had a detectability limit of 50 pg per assay tube. Using duplicate methanol extract residues enabled us to assess the measurement precision of the RIA step of the analysis. Repeatability of total corticosterone concentration per feather was 0.69 (F_104;105_ = 5.4, P<0.0001).

### Statistics

Pre-treatment values of studied variables were analysed using one-way ANOVA, with treatment as a single factor with four levels (LPS−FEAR−, LPS−FEAR+, LPS+FEAR−, LPS+FEAR+) in order to test for possible differences between treatment groups prior to the manipulations. Average parameter values per treatment group and ANOVA statistics are presented in Table S2. Treatment effects on changes in measured parameters between the first and second blood sampling were tested using repeated measures ANOVA. Full models with both treatments (LPS and FEAR) and bird age (yearling vs older) as factors are presented in Table S3. Final models, containing only significant predictors (if any were found), are presented in Table 1. In the case of feather corticosterone, the first measurement represents the corticosterone content of wild-grown feathers while the second measurement represents the corticosterone content of lab-grown feathers. In the cases of DNA and protein damage and BA titres, only the effects of treatments on post-treatment values could be analyzed using ANOVA. The assumptions of parametric tests (normality of residuals, homogeneity of variances) were met for all models. All tests were two-tailed with an α-level of 0.05. Sample sizes varied between analyses due to our inability to collect sufficient good quality blood samples from all birds.

**Table 1:** Effects of LPS and predator image exposure (FEAR) treatments on changes (or final values) of physiological parameters of captive greenfinches between the 15^th^ and 23^rd^ day of experiment.

Dependent variable	Predictor	F df	P
Mass	TIME	236.34 1,65	<0.0001
Total plasma protein	LPS	21.78 1,32	0.0001
	TIME	50.58 1,32	<0.0001
	TIME*LPS	17.84 1,32	0.0002
Glutathione	LPS	0.01 1,57	<0.0001
	TIME	27.29 1,57	<0.0001
	TIME*LPS	13.04 1,57	0.0006
TAC	TIME	0.66 1,33	0.42
OXY	LPS	5.74 1,22	0.026
	TIME	11.96 1,22	0.002
	TIME*LPS	13.64 1,22	0.001
Uric acid	TIME	0.17 1,34	0.69
Feather corticosterone	TIME	6.67 1,50	0.013
	TIME*LPS	6.25 1,50	0.016
DNA damage	LPS	0.08 1,52	0.78
	FEAR	1.89 1,52	0.18
	LPS*FEAR	0.58 1,52	0.45
Carbonylated proteins	LPS	0.39 1,22	0.54
	FEAR	2.08 1,22	0.16
	LPS*FEAR	1.59 1,22	0.22
Oocyst count	TIME	2.19 1,65	0.14
B. arbortus antibody titres	LPS	0.48 1,52	0.49
	FEAR	0.16 1,52	0.69
	LPS*FEAR	<0.001 1,52	0.96

For DNA damage and carbonylated proteins, only final values measured on day 23 of the experiment are analysed with ANOVAs. For the *B. arbortus* antibody titres, values measured on day 31 are analysed with ANOVA. For the rest of variables, pre- vs post-treatment values are analysed in repeated measures ANOVA-s with TIME as a repeated measure (pre- vs post-treatment chage). For repeated measures models, only significant effects (if present) are given. Full repeated measures models are presented in Table S3.

## Results

**Figure 3:**
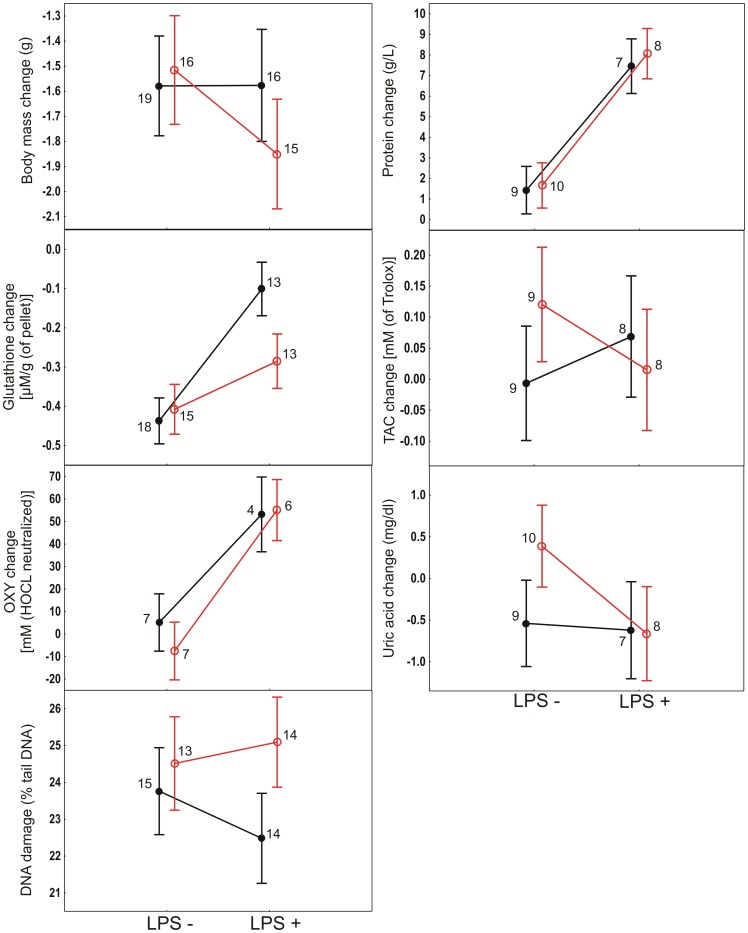
Average±SE changes in physiological variables between the 15^th^ and 23^rd^ day of the experiment among the LPS-injected (‘LPS +’) and saline-injected (‘LPS −’) greenfinches. Filled symbols represent birds not exposed to a predator image, open symbols represent birds exposed to a predator image. Numbers indicate sample sizes for respective treatment groups.

**Figure 4:**
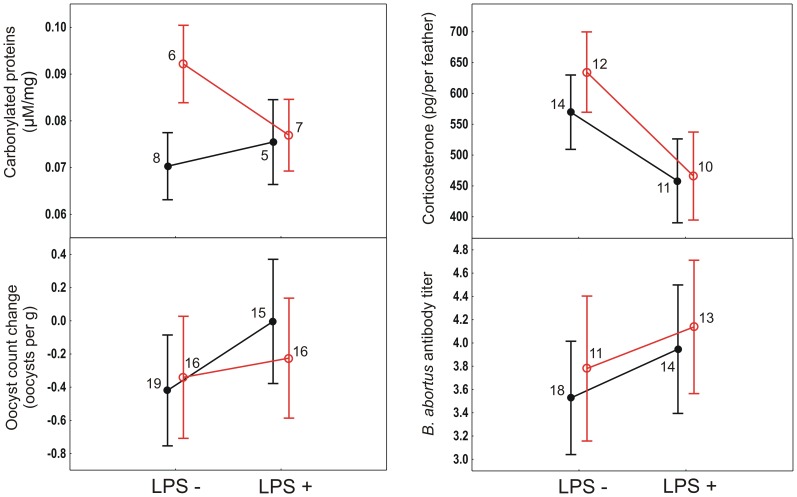
Average±SE changes in coccidian oocyst load in greenfinch faecal samples between the pre-treatment and post-treatment period, protein carbonyls on the 15^th^ day of the experiment, BA antibody titre and corticosterone content of feathers grown during the experiment. Legend as in Fig. 3.

None of the studied variables differed between treatment groups before the experimental manipulations (Table S2). Injection of LPS significantly increased plasma protein and erythrocyte glutathione levels and one measure of plasma antioxidant capacity (OXY) (Table 1, Fig. 3). Compared with saline-injected birds, LPS-injected birds deposited less corticosterone into tail feathers grown during the experiment (Table 1, Fig. 4). None of the other measured haematological parameters were affected by the LPS injection (Table 1, Figs 3 – 4). LPS injection did not affect body mass dynamics between the first and second blood sampling (Table 1, Fig. 3). However, comparison of bird body mass at the time of the LPS or saline injection on day 21 and 72 h later on day 24 revealed a significantly greater decrease in LPS-injected (1.59±0.99 (SD) g, n = 31) than saline-injected birds (0.67±0.87 (SD) g, n = 35; t = 4.0, P = 0.0002). Exposure to the predator image and bird age did not affect any of the measured parameters (Table 1, Table S3).

## Discussion

### Effects of endotoxin on redox homeostasis

The primary aim of the current study was to establish the costs of inflammatory responses in terms of disruption to redox homeostasis and trade-offs with other components of immunity. Our assessment of the first component was partially successful, as we detected significant changes in two parameters related to redox status, i.e., erythrocyte glutathione levels and plasma OXY.

Glutathione (GSH) is the major endogenous antioxidant, participating directly in the neutralization of free radicals and recycling exogenous antioxidants such as vitamins C and E. GSH is a cofactor in GPx enzymes, which reduce H_2_O_2_ and peroxidized fatty acid residues, and is involved in detoxification of xenobiotics and in many aspects of immunomodulation. It also plays a fundamental role in numerous metabolic and biochemical reactions, such as DNA synthesis and repair, regulation of apoptosis, protein synthesis, prostaglandin synthesis, amino acid transport and enzyme activation [54], [60], [61].

To our knowledge, this is the first study to detect an increase in glutathione in response to immune system activation in a wild bird species. Furthermore, despite the widespread practice of using LPS for induction of inflammation and OS in poultry, we were unable to find any studies where the effects of LPS on erythrocyte GSH had been measured. However, a reduction in the activity of the glutathione peroxidise (a GSH-dependent enzyme) in response to intravenous injection of *Salmonella enteriditis* LPS in the serum of chickens has been recorded [25]. The same study also detected a decrease in the activity of another antioxidant enzyme (superoxide dismutase) and an increase in plasma malondialdehyde (a marker of lipid peroxidation) in the serum of LPS-injected chickens. In Fischer rats, *S. enteritidis*, LPS dose dependently increased plasma GSH [62]. In another study of rats, a significant increase in liver and plasma total GSH was observed 5 h after endotoxin treatment (acute phase), followed by a decrease of these parameters below control values at 48 h (recovery phase) [63]. These findings support the hypothesis that increased erythrocyte GSH levels in the current study reflect the compensatory up-regulation of GSH synthesis among immune-challenged birds.

Another parameter related to redox homeostasis that was sensitive to LPS treatment was plasma OXY. We are aware of four previous studies where this marker has been measured in relation to immune challenge. In Eurasian kestrel (*Falco tinnunculus*) nestlings, immunostimulation with a plant lectin phytohaemagglutinin (PHA) reduced serum OXY levels over 24 h [64]. Diamond doves (*Geopelia gunerata*), immunised with sheep erythrocytes also had significantly lower plasma OXY six days after immunisation, when primary antibody production reaches its maximum [65]. Similarly, a non-significant tendency (P = 0.08) for reduced OXY was detected in a study of homing pigeons (*Columba livia*) 18 h after LPS injection [66], while LPS injection had no effect on plasma OXY in the nestlings of European starlings (*Sturnus vulgaris*) over 24 h [26]. Our results are in contrast with the above findings. We suggest that one of the reasons for this discrepancy might be the timing of blood sampling with respect to immunisation. In the current study, birds were bled ca 38 h after LPS injection, which is a longer interval than those used in the above-cited studies. It thus appears possible that the results obtained in the current study characterise the phase of compensatory up-regulation of antioxidant protection mechanisms that follows initial production of pro-oxidant compounds when inflammatory response is initiated (see also [66]).

The lack of an effect of LPS injection on plasma TAC and uric acid is consistent with the results of Cohen and colleagues [67], obtained from domestic chickens (sampled at 2, 7, 12 and 14 h PI). Another study in chickens, however, detected a decrease in plasma uric acid levels two days after LPS injection [68].

We are not aware of previous studies measuring DNA or protein damage in response to LPS administration in birds. In mice, LPS injection can increase carbonylation of plasma proteins [69]. It is possible that carbonylated proteins of avian erythrocytes are not a sufficiently sensitive marker to detect oxidative damage. For instance, we also failed to detect an increase in protein carbonyls in response to paraquat-induced OS in greenfinches [70]. The absence of effects of LPS on DNA damage in the current study could possibly be ascribed to our inability to measure oxidized purines, as the current version of the assay quantifies DNA strand breaks and alkali-labile sites only. These parameters cannot be specifically related to oxidation, since they result from various forms of damage, and might also represent intermediates in the repair process [56]. It should be noted, however, that a similar alkaline comet assay was able to detect a relationship between DNA damage, ornamentation and survival in common yellowthroats (*Geothlypis trichas*) [71]. In budgerigars (*Melopsittacus undulatus*), a similar assay revealed that a diet based on reduced levels of antioxidants led to increased DNA damage [72]. It is thus plausible that the absence of effects of LPS on our markers of oxidative damage was genuine or that the 38 h interval between the LPS injection and blood sampling was too long for detection of rapid and transient changes in oxidative damage. In the context of findings that LPS injection affects body mass for at least 72 h PI and plasma total protein content 38 h PI, one could conclude that DNA integrity is well protected from such inflammatory insults. Alternatively, quick repair of DNA may be highly prioritized as compared to restoration of body mass, removal of positive acute phase proteins from blood or restoration of blood volume (if increased protein levels result from a fever-induced dehydration [26]).

### Endotoxin, corticosterone and immune function

Perhaps the most surprising result of the current study was that LPS-injected birds deposited less corticosterone into their feathers during the experiment than saline-injected birds. Both the acute phase response and avian stress response share some of the same neuroendocrine control mechanisms, such as the HPA-axis [39], and endotoxin has been referred to as an ‘immunological stressor’ [37], [41]. Indeed, LPS administration experiments typically document a rise in blood corticosterone levels, lasting from minutes to several hours following injection [39] but see [44].

Feather corticosterone reflects plasma hormone levels during the period of feather growth and thus integrates variation in the baseline level, the magnitude of the stimulated response, the time course for the stress response and the number of stressors experienced [57], [58]. We therefore expected that the LPS-induced surge of corticosterone would be reflected in higher feather corticosterone levels among LPS-treated birds. However, exactly the opposite was found. A possible explanation for such a result could be stress-induced downregulation of corticosterone production due to a negative feedback mechanism (reviewed by [73]). For instance, chronic psychological stress [74], feather clipping [75] and corticosterone administration [76] have been shown to suppress baseline and/or stress-induced corticosterone levels in birds. Such down-regulation of the endogenous stress response can be relatively long-lasting; for example, up to 20 days in kestrel *Falco tinnunculus* nestlings [76]. The adaptive explanation for this phenomenon would be protection of an organism from the pathological effects (immune suppression, energy dysregulation, neuronal cell death, behavioural impairment) of chronically elevated corticosterone levels [73], [77].

We are unaware of any study reporting long-lasting effects of LPS administration on the functionality of the HPA-axis. However, we cannot currently provide any alternative explanation for the reduced feather corticosterone of LPS-injected birds besides deactivation, impairment or down-regulation of corticosterone secretion cascade due to the endotoxin-induced immune response. Long-lasting consequences of inflammatory responses have also been recorded in a previous study of greenfinches, where heterophil concentration (a haematological index of stress and/or inflammation) remained elevated for at least 30 days after administration of phytohaemagglutinin [78]. We propose that activation of the immune system with pro-inflammatory antigens can induce long-lasting changes in the functionality of the HPA axis that resemble the effects of chronic stress and eventually affect the stress responsiveness of birds. This hypothesis would be testable by measuring feather corticosterone levels subsequent to up-and down-regulation of HPA axis activity and long-term responses to different antigens.

None of the treatments affected the antibody response to *Brucella abortus* antigen injection. In mammals, antibodies produced against BA are predominantly IgG2a, which are characteristic of Th1-cell mediated (i.e. inflammatory) immune responses [79]. Assuming a similar mechanism in birds, high antibody titres can be interpreted as strong inducers of Th1 helper cell mediated immunity. Th1 type immune responses are typically down-regulated by the major stress hormones, i.e., glucocorticoids and catecholamines [10], [80]. This scenario was confirmed in previous studies in greenfinches [81] and great tits (*Parus major*) [82], which indicated that BA antibody titres are lower in stressed individuals. The lack of an effect of predator image exposure on immune response to BA in the current study can probably be explained by the generally mild nature of this treatment (see below). However, we lack an explanation of why BA antibody titres were not affected by LPS administration.

Similarly to the BA-response, we found no evidence that our treatments affected the intensity of chronic coccidiosis. A possible explanation for this pattern could be tolerance of greenfinches to their naturally acquired coccidian strains [46], which would be manifested by maintenance of immune responses against chronic infection at a low level, such that these are not sensitive to external manipulations of the immune system. Similarly, intensity of chronic coccidiosis was unaffected by immune challenge with sheep erythrocytes [18]. However, injection of an inflammatory agent, phytohaemagglutinin, was found to suppress the intensity of chronic coccidiosis, but only among the carotenoid-supplemented greenfinches [83]. Hence, interactions between processes affecting novel challenges and resistance to chronic infections appear complex.

### Effects of predator image exposure

Contrary to our predictions, we did not find any evidence that exposing birds to the image of a predator amplified the effects of physiological stress induced by endotoxin. We had reason to expect a physiological impact of predator image exposure because long-term effects of stressful events, such as capture [84], nest predation [27], translocation [85], restraint [86] or high stocking density [30], on different behavioural and physiological parameters have been recorded in birds. Furthermore, we detected that exposure to a predator image reduced total locomotor activity in greenfinches for at least 44 h after the treatment [87]. On the other hand, predator image exposure (unlike endotoxin) had no effect on deposition of corticosterone into feathers grown during the experiment. The latter finding implies that our treatment had a relatively mild stressful impact.

### Conclusions

This experiment demonstrated up-regulation of two markers of antioxidant protection – erythrocyte glutathione and plasma OXY in response to immune challenge with bacterial endotoxin. These findings suggest that inflammatory responses alter redox homeostasis. It is possible that such up-regulation of antioxidant protection *per se* is costly in terms of some ecologically relevant currency [60], [70], as otherwise it would be difficult to explain why animals do not permanently maintain high levels of GSH and plasma antioxidants. Establishing such potential costs of up-regulation of antioxidant protection machinery is a challenge for future studies in the realm of oxidative stress ecology.

## Supporting Information

Table S1 **Number of greenfinches subjected to FEAR and LPS treatments with respect to age (yearling vs older).**
(DOC)

Table S2 **Average pre-treatment values of physiological parameters of captive greenfinches injected with LPS or exposured to predator image.**
(DOC)

Table S3 **Effects of LPS and predator image exposure treatments and age of birds on changes of physiological parameters of captive greenfinches between the 15^th^ and 23^rd^ day of experiment in repeated measures ANOVA-s.**
(DOC)
