# Supplementary material for: Effects of Endotoxin and Psychological Stress on Redox Physiology, Immunity and Feather Corticosterone in Greenfinches
Source: PLoS One. 2013 Jun 21;8(6):e67545. doi: 10.1371/journal.pone.0067545 (PMC3689720; doi:10.1371/journal.pone.0067545)
Supplement: Table S1 — Number of greenfinches subjected to FEAR and LPS treatments with respect to age (yearling vs older). (DOC) [file pone.0067545.s001.doc]

Table S1. Number of greenfinches subjected to FEAR and LPS treatments with respect to age (yearling vs older).

| LPS | FEAR | age | N |
| --- | --- | --- | --- |
| - | - | 2 | 10 |
| - | - | 1 | 9 |
| - | + | 2 | 9 |
| - | + | 1 | 7 |
| + | - | 2 | 9 |
| + | - | 1 | 6 |
| + | + | 2 | 9 |
| + | + | 1 | 7 |
