# Supplementary material for: Effects of Endotoxin and Psychological Stress on Redox Physiology, Immunity and Feather Corticosterone in Greenfinches
Source: PLoS One. 2013 Jun 21;8(6):e67545. doi: 10.1371/journal.pone.0067545 (PMC3689720; doi:10.1371/journal.pone.0067545)
Supplement: Table S2 — Average pre-treatment values of physiological parameters of captive greenfinches injected with LPS or exposured to predator image. (DOC) [file pone.0067545.s002.doc]

Table S2. Average pre-treatment values of physiological parameters of captive greenfinches injected with LPS or exposured to predator image (FEAR). Presence of possible between-group differences is tested in one-way ANOVA with treatment as a factor with four levels.

| Dependent variable | Treatment | Mean | SD | N | F _df_ | p |
| --- | --- | --- | --- | --- | --- | --- |
| Mass (g) | Fear - / LPS - | 28.45 | 2.14 | 19 | 0.1 _3,62_ | 0.96 |
|  | Fear + / LPS - | 28.38 | 1.50 | 16 |  |  |
|  | Fear - / LPS + | 28.31 | 1.37 | 15 |  |  |
|  | Fear + / LPS + | 28.15 | 1.26 | 16 |  |  |
|  |  |  |  |  |  |  |
| Proteins (g/L) | Fear - / LPS - | 21.80 | 2.97 | 11 | 0.62 _3,36_ | 0.61 |
|  | Fear + / LPS - | 22.11 | 3.07 | 11 |  |  |
|  | Fear - / LPS + | 23.18 | 2.20 | 10 |  |  |
|  | Fear + / LPS + | 22.86 | 1.50 | 8 |  |  |
|  |  |  |  |  |  |  |
| Glutathione (μmol/mg) | Fear - / LPS - | 1.54 | 0.51 | 19 | 1.25 _3,59_ | 0.30 |
|  | Fear + / LPS - | 1.50 | 0.39 | 15 |  |  |
|  | Fear - / LPS + | 1.40 | 0.28 | 15 |  |  |
|  | Fear + / LPS + | 1.28 | 0.35 | 14 |  |  |
|  |  |  |  |  |  |  |
| TAC (mM) | Fear - / LPS - | 0.41 | 0.28 | 9 | 0.05 _3,59_ | 0.99 |
|  | Fear + / LPS - | 0.38 | 0.37 | 11 |  |  |
|  | Fear - / LPS + | 0.40 | 0.30 | 9 |  |  |
|  | Fear + / LPS + | 0.36 | 0.25 | 10 |  |  |
|  |  |  |  |  |  |  |
| OXY (mM) | Fear - / LPS - | 187.64 | 15.90 | 9 | 0.7 _3,26_ | 0.56 |
|  | Fear + / LPS - | 208.31 | 36.62 | 10 |  |  |
|  | Fear - / LPS + | 197.00 | 43.42 | 5 |  |  |
|  | Fear + / LPS + | 197.97 | 26.70 | 6 |  |  |
|  |  |  |  |  |  |  |
| Uric acid (mg/dL) | Fear - / LPS - | 3.76 | 1.02 | 11 | 1 _3,37_ | 0.41 |
|  | Fear + / LPS - | 4.08 | 1.29 | 11 |  |  |
|  | Fear - / LPS + | 3.56 | 1.53 | 10 |  |  |
|  | Fear + / LPS + | 3.12 | 1.18 | 9 |  |  |
|  |  |  |  |  |  |  |
| Wild feather corticosterone  (pg/feather) | Fear - / LPS - | 603.87 | 194.25 | 16 | 1.26 _3,54_ | 0.30 |
|  | Fear + / LPS - | 567.89 | 179.72 | 15 |  |  |
|  | Fear - / LPS + | 611.70 | 143.72 | 13 |  |  |
|  | Fear + / LPS + | 686.46 | 142.62 | 14 |  |  |
|  |  |  |  |  |  |  |
| Oocyst count ( ln(oocysts/g)) | Fear - / LPS - | 10.11 | 1.52 | 19 | 1.11 _3,62_ | 0.35 |
|  | Fear + / LPS - | 9.12 | 1.81 | 16 |  |  |
|  | Fear - / LPS + | 9.98 | 1.74 | 15 |  |  |
|  | Fear + / LPS + | 9.71 | 1.79 | 16 |  |  |
