# Supplementary material for: Effects of Endotoxin and Psychological Stress on Redox Physiology, Immunity and Feather Corticosterone in Greenfinches
Source: PLoS One. 2013 Jun 21;8(6):e67545. doi: 10.1371/journal.pone.0067545 (PMC3689720; doi:10.1371/journal.pone.0067545)
Supplement: Table S3 — Effects of LPS and predator image exposure treatments and age of birds on changes of physiological parameters of captive greenfinches between the 15th and 23rd day of experiment in repeated measures ANOVA-s. (DOC) [file pone.0067545.s003.doc]

Table S3. Effects of LPS and predator image exposure (FEAR) treatments and AGE of birds (yearling vs older) on changes of physiological parameters of captive greenfinches between the 15^th^ and 23^rd^ day of experiment in repeated measures ANOVA-s. TIME is a repeated measure (pre- vs post-treatment). For each model, between-group statistics are presented above the line and within-individual statistics below the line.

| Dependent variable | Predictor | F _df_ | P |
| --- | --- | --- | --- |
| Mass | LPS | 0.39 _1,60_ | 0.53 |
|  | FEAR | 0.25 _1,60_ | 0.62 |
|  | AGE | 0 _1,60_ | 0.99 |
|  | LPS*FEAR | 0.14 _1,60_ | 0.71 |
|  | LPS*AGE | 0.73 _1,60_ | 0.40 |
|  | TIME | 223.79 _1,60_ | <0.001 |
|  | TIME*LPS | 0.99 _1,60_ | 0.32 |
|  | TIME*FEAR | 0.3 _1,60_ | 0.58 |
|  | TIME*AGE | 0.83 _1,60_ | 0.37 |
|  | TIME*LPS*FEAR | 0.83 _1,60_ | 0.37 |
|  | TIME*LPS*AGE | 0.78 _1,60_ | 0.38 |
|  |  |  |  |
| Proteins | LPS | 17.91 _1,28_ | <0.001 |
|  | FEAR | 0.02 _1,28_ | 0.90 |
|  | AGE | 0.06 _1,28_ | 0.81 |
|  | LPS*FEAR | 0 _1,28_ | 0.97 |
|  | LPS*AGE | 0.3 _1,28_ | 0.59 |
|  | TIME | 44.53 _1,28_ | <0.001 |
|  | TIME*LPS | 17.23 _1,28_ | <0.001 |
|  | TIME*FEAR | 0.35 _1,28_ | 0.56 |
|  | TIME*AGE | 0.27 _1,28_ | 0.61 |
|  | TIME*LPS*FEAR | 0.02 _1,28_ | 0.90 |
|  | TIME*LPS*AGE | 1.55 _1,28_ | 0.22 |
|  |  |  |  |
| Glutathione | LPS | 0.07 _1,53_ | 0.79 |
|  | FEAR | 1.54 _1,53_ | 0.22 |
|  | AGE | 1.3 _1,53_ | 0.26 |
|  | LPS*FEAR | 0.98 _1,53_ | 0.33 |
|  | LPS*AGE | 0.4 _1,53_ | 0.53 |
|  | TIME | 25.06 _1,53_ | <0.001 |
|  | TIME*LPS | 11.33 _1,53_ | <0.001 |
|  | TIME*FEAR | 0 _1,53_ | 0.97 |
|  | TIME*AGE | 0.12 _1,53_ | 0.73 |
|  | TIME*LPS*FEAR | 0.52 _1,53_ | 0.47 |
|  | TIME*LPS*AGE | 0.01 _1,53_ | 0.91 |
|  |  |  |  |
| TAC | LPS | 0 _1,28_ | 0.95 |
|  | FEAR | 0 _1,28_ | 0.99 |
|  | AGE | 0.01 _1,28_ | 0.92 |
|  | LPS*FEAR | 0.19 _1,28_ | 0.66 |
|  | LPS*AGE | 0 _1,28_ | 0.96 |
|  | TIME | 0.14 _1,28_ | 0.71 |
|  | TIME*LPS | 0.25 _1,28_ | 0.62 |
|  | TIME*FEAR | 0.4 _1,28_ | 0.53 |
|  | TIME*AGE | 1.31 _1,28_ | 0.26 |
|  | TIME*LPS*FEAR | 0.49 _1,28_ | 0.49 |
|  | TIME*LPS*AGE | 0.63 _1,28_ | 0.43 |
|  |  |  |  |
| OXY | LPS | 4.01 _1,18_ | 0.06 |
|  | FEAR | 0.32 _1,18_ | 0.58 |
|  | AGE | 0.52 _1,18_ | 0.48 |
|  | LPS*FEAR | 0.02 _1,18_ | 0.88 |
|  | LPS*AGE | 0.19 _1,18_ | 0.67 |
|  | TIME | 9.2 _1,18_ | 0.01 |
|  | TIME*LPS | 11.8 _1,18_ | <0.001 |
|  | TIME*FEAR | 0.18 _1,18_ | 0.68 |
|  | TIME*AGE | 1.07 _1,18_ | 0.31 |
|  | TIME*LPS*FEAR | 0.51 _1,18_ | 0.48 |
|  | TIME*LPS*AGE | 0.04 _1,18_ | 0.83 |
|  |  |  |  |
| Uric acid | LPS | 1.55 _1,29_ | 0.22 |
|  | FEAR | 0.16 _1,29_ | 0.69 |
|  | AGE | 0.19 _1,29_ | 0.67 |
|  | LPS*FEAR | 0.31 _1,29_ | 0.58 |
|  | LPS*AGE | 0.02 _1,29_ | 0.90 |
|  | TIME | 1.22 _1,29_ | 0.28 |
|  | TIME*LPS | 0.61 _1,29_ | 0.44 |
|  | TIME*FEAR | 4.1 _1,29_ | 0.05 |
|  | TIME*AGE | 6.68 _1,29_ | 0.02 |
|  | TIME*LPS*FEAR | 0.11 _1,29_ | 0.74 |
|  | TIME*LPS*AGE | 0.51 _1,29_ | 0.48 |

|  |  |  |  |
| --- | --- | --- | --- |
| Feather corticosterone | LPS | 0.64 _1,46_ | 0.43 |
|  | FEAR | 0.33 _1,46_ | 0.57 |
|  | AGE | 0.05 _1,46_ | 0.82 |
|  | LPS*FEAR | 0.04 _1,46_ | 0.85 |
|  | LPS*AGE | 0.79 _1,46_ | 0.38 |
|  | TIME | 5.68 _1,46_ | 0.02 |
|  | TIME*LPS | 5.83 _1,46_ | 0.02 |
|  | TIME*FEAR | 0.04 _1,46_ | 0.85 |
|  | TIME*AGE | 0.41 _1,46_ | 0.52 |
|  | TIME*LPS*FEAR | 1.45 _1,46_ | 0.24 |
|  | TIME*LPS*AGE | 0.01 _1,46_ | 0.93 |
|  |  |  |  |
| Oocyst count | LPS | 0.8 _1,60_ | 0.38 |
|  | FEAR | 2.21 _1,60_ | 0.14 |
|  | AGE | 4.44 _1,60_ | 0.04 |
|  | LPS*FEAR | 0.3 _1,60_ | 0.59 |
|  | LPS*AGE | 0 _1,60_ | 0.99 |
|  | TIME | 1.96 _1,60_ | 0.17 |
|  | TIME*LPS | 0.29 _1,60_ | 0.59 |
|  | TIME*FEAR | 0 _1,60_ | 0.99 |
|  | TIME*AGE | 0 _1,60_ | 0.98 |
|  | TIME*LPS*FEAR | 0.26 _1,60_ | 0.61 |
|  | TIME*LPS*AGE | 1.14 _1,60_ | 0.29 |
